# Caregivers’ understanding of childhood influenza vaccination during the epidemic in China. A mixed-methods study

**DOI:** 10.3389/fpubh.2023.1196019

**Published:** 2023-08-10

**Authors:** Kaiyi Han, Zhiyuan Hou, Shiyi Tu, Qian Wang, Simeng Hu, Yuting Xing, Jing Du, Shujie Zang, Tracey Chantler, Heidi Larson

**Affiliations:** ^1^School of Public and Health, Fudan University, Shanghai, China; ^2^Department of Infectious Disease Epidemiology, London School of Hygiene & Tropical Medicine, London, United Kingdom; ^3^NHC Key Laboratory of Health Technology Assessment, Fudan University, Shanghai, China; ^4^Department of Global Health and Development, London School of Hygiene and Tropical Medicine, London, United Kingdom; ^5^Department of Health Metrics Sciences, University of Washington, Seattle, WA, United States

**Keywords:** influenza, vaccine, mixed-methods, China, child

## Abstract

**Introduction:**

Influenza vaccination uptake among young children has been poor in China, but it is unclear how it changed during the COVID-19. This study aimed to investigate the uptake status and reasons of childhood influenza vaccination during the pandemic in China.

**Methods:**

A mixed-methods study combining a questionnaire survey and semi-structured interviews was conducted in Anhui, Shaanxi, and Guangdong provinces between September and November 2021. 2081 caregivers completed the valid questionnaire. 38 caregivers participated in interviews, and data were analyzed thematically, using deductive and inductive coding.

**Results:**

A total of 2081 caregivers completed the valid questionnaire, and 38 caregivers participated in interviews. Among the caregivers, a total of 1796 were in the age group for high-risk groups in the 2019–2020 flu season, and 46.10% reported that their children received influenza vaccination in the 2019–2020 flu season; 43.63% said that they vaccinated their children against influenza in the 2020–2021 flu season. Many caregivers indicated that the adoption of nonpharmacologic interventions (NPIs) during COVID-19 reduced the risk of influenza infection for children. Most caregivers consider the severity of influenza to be low, and some confused the common cold with influenza. Meanwhile, some caregivers lack confidence in the vaccine’s effectiveness and importance. They thought that vaccines are not effective in preventing the constantly mutating virus. Despite clear perceptions about the severity of influenza and the effectiveness of the vaccine, we found that most caregivers did not receive any relevant medical information, and the communication about vaccines between caregivers and professional information sources, such as healthcare workers, is inadequate. Hence, caregivers have no scientific evidence to back up their perceptions. In terms of access to vaccination service, caregivers reported conflicts between time of vaccination service and their schedule, and the need for vaccine prices to be reduced.

**Discussion:**

Targeted interventions are needed to address caregivers’ lack of risk perception on influenza during COVID-19 and promote communication between caregivers and professional information sources. Extending vaccination service hours and increasing the number of vaccine clinics close to residential areas and expansion of financing sources for self-paid vaccination could facilitate the access to influenza vaccination service.

## 1. Introduction

The COVID-19 was declared a pandemic by the WHO on March 11, 2020 (1), and continues to have a major impact worldwide in 2021 (2). Co-circulation of COVID-19 and influenza may lead to great burden on hospitalization and intensive care unit (ICU) resources (3). To avoid potential surge resource needs, it’s essential to focus on maximizing the impact of the available control measures for both COVID-19 and influenza.

Influenza vaccination of risk groups should be the cornerstone of seasonal influenza management (4). Recommended by the World Health Organization (WHO), vaccination uptake among high-risk groups, is an effective strategy for decreasing influenza burden and therefore allowing for better preparedness for anticipated COVID-19 waves. In China, however, influenza vaccine remains excluded from national Expanded Program of Immunization (EPI) and needs to be paid out of pocket (5), although many countries have included it in their National Immunization Program (6–10).

Influenza vaccination uptake has been keeping at low level for decades in China and difference exists between regions, even for children aged 6–59 months-one of the high-risk groups recommended by the WHO (11). Uptake rate of influenza vaccination among the young children ranged from 3.1% in a city in Fujian Province in 2015 to 47.7% in Guangzhou city in 2013 (12–14). The low influenza vaccination coverage cannot protect children during the pandemic. Studies have shown that caregivers’ knowledge, perceived risk of getting influenza, beliefs regarding the vaccine’s efficacy and safety, perceived barriers of vaccination and recommendation from healthcare workers (HCWs) were associated with their decision on childhood influenza vaccination (12–14). An important barrier, leading to doubts about the trade-offs between the benefits and risks of vaccination, is a lack of appropriate information (15). Sufficient communication from professional information sources could enhance their capacity to counter negative information about vaccines and achieve community support for vaccination programs.

The Strategic Advisory Group of Experts on Immunization (SAGE) established in 2012, concluded that communication can play an important role in the public’s decision to vaccinate (16). In most settings, communication about childhood vaccination is common, but to date, there have been few attempts to explore how caregivers perceive and experience communication about vaccination and if the information or mode of communication influences their intention to vaccinate (17). In addition, the COVID-19 pandemic may affect the public’s perception on influenza in different ways. Due to increased diversion of resources to COVID-19, there is a heightened importance for seasonal influenza vaccination to minimize the viral reservoir in the population (3). On the other hand, continued use of face coverings and reinstating local lockdowns during periods of increased transmission could substantially reduce the rates of infection for both COVID-19 and influenza diseases (18). A study has shown decreased influenza incidence in 2020 after adoption of nonpharmacologic interventions (NPIs) as compared with prior seasons (19). Thus, it is necessary to understand how public perceptions of influenza vaccination changed during the COVID-19 pandemic. This study is part of the vaccine confidence survey in China. Our published study focused on the quantitative analysis of the association between childhood influenza vaccination and caregivers’ perceived susceptibility and severity of influenza, perceived benefits and barriers to influenza vaccination, the influence of different information sources and caregivers’ emotions (20). In this study, we further explores reasons behind caregivers’ perceptions of influenza and influenza vaccine, and also their communication and interaction with different information sources. This will help understand not only the reasons for caregivers’ perception about influenza and vaccines, but also how caregivers perceive and understand the communication on influenza vaccines, and whether and how this influences their decision to vaccinate, to furtherly contribute to structure and implement communication interventions appropriately.

## 2. Methods

### 2.1. Study design and sites

We conducted a mixed-methods cross-sectional study across three purposefully selected Chinese provinces, between September to November 2021. China has 34 provinces, cities, autonomous regions, and special administrative regions with wide regional inequality. We purposefully selected Guangdong (South, ranked 6th in the 2020 provincial GDP ranking of economic development), Anhui (Central-East, ranked 15th) provinces, and Shaanxi (Northwest, ranked 19th). Multistage stratified random cluster sampling was conducted in three stages to enroll eligible participants, prefecture-level cities, urban and rural areas, and local sampling sites: vaccination clinics (age 0.5–3), and kindergartens (age 3–5). (1) We select one urban district from Shenzhen megacity, Guangdong province, and one urban district and one rural county from Anhui and Shanxi provinces; (2) three or four communities were selected according to their socioeconomic status in each district/county. (3) In each sampled community, one vaccination clinic and/or one kindergarten were selected to recruit caregivers. Every vaccination clinic and kindergarten in the selected communities had an equal chance of selection.

The Fudan University School of Public Health, and the London School of Hygiene & Tropical Medicine Ethics committees approved the study protocol [FDU IRB#2018–10–0703, LSHTM Ethics Ref 16016].

### 2.2. Survey data collection and analysis

#### 2.2.1. Recruitment of survey participant

The survey participants were defined as caregivers (parents and guardians) of children aged six and under. In each sampled community, eligible participants were recruited from one vaccination clinic and one kindergarten, respectively. Caregivers of all children visiting the vaccination clinics on a given day during the survey period and from a class in the sampled kindergartens were invited to participate in the survey. Caregivers were invited to complete an online questionnaire after signing informed consent. Data from the self-completed online questionnaires were automatically uploaded to the Wenjuanxing online platform in real time.

In total 2081 caregivers were surveyed, 360 from Nanshan district in Guangdong Province, 360 and 465 were from Dongzhi County and Shushan district in Anhui Province, 423 and 473 were from Jingyang county and Qindu district in Shaanxi Province, respectively.

#### 2.2.2. Survey questionnaire

The questionnaire included questions about demographic and socio-economic characteristics, and childhood influenza vaccination. Childhood influenza vaccination behavior was measured with two questions, “Did your child receive influenza vaccine during the last flu season (October 2020 March 2021)?” and “Did your child receive influenza vaccine during the 2019–2020 flu season?”

#### 2.2.3. Statistical analysis for survey data

Descriptive analyses were performed to compare the levels of childhood influenza vaccine uptake by living area and child’s age during the two flu seasons. All statistical analyses were performed using Stata, version 14.0 (StataCorp LP, College Station, TX, USA).

### 2.3. Interview data collection and analysis

#### 2.3.1. Recruitment of interview participant

We recruited caregivers for interview from the vaccination clinic in each sampled community. We applied purposive sampling to obtain a range of perspectives and achieve variation in terms of childhood influenza vaccination status. Caregivers were sampled according to their children’s influenza vaccination status in the 2020–2021 flu season, i.e., whether they (i) vaccinated their children against influenza in the 2020–2021 flu season, or (ii) did not vaccinate their children. For each influenza vaccination status, one or two caregivers were recruited from one vaccination clinic in each sampled community. All participants signed a consent form before the interview commenced and were given a token. (e.g., cup) for their time and effort.

In total 38 caregivers were interviewed: 8 from Nanshan district in Guangdong province, 8 and 10 from Dongzhi county and Shushan district in Anhui province, and 5 and 7 from Jingyang county and Qingdu district in Shaanxi province.

#### 2.3.2. Interview guides

We interviewed a total of 38 caregivers at vaccination clinics from September to November 2021. Each interview lasted approximately 20 min. The sample of interviewees was determined by the principal of data saturation (no new information emerges from the interviews). We developed interview guides according to the Hesitancy Determinants Matrix (16). Key areas explored in the research included:

- Access to influenza vaccination service, including the transportation to the vaccine clinics, the time of the vaccination service, satisfaction with HCWs, and affordability of influenza vaccines.
- Perception of the disease and the vaccine, including perceived causes, symptoms, and complications of influenza, their perceived susceptibility and severity of influenza of children before and during COVID-19, confidence in influenza vaccines’ importance, effectiveness, and safety.
- Sources of information and influencers in relation to influenza vaccination.
- Caregivers’ communication with professional information sources, including HCWs and staffs from Center for Disease Control and Prevention (CDCs) or Community Healthcare Centers (CHCs).

The interviews were audio-recorded (with participant consent) and transcribed verbatim by a professional software service (iflynote) (21). We also took notes during the sessions. All participants were informed that participation was voluntary and that they could withdraw at any time. All interviewees were assured of the confidentiality of the interviews. None of the participants left the study before the interviews were concluded.

#### 2.3.3. Data analysis for interviews

All interviews were audio-recorded and transcribed verbatim by the research team. A thematic analysis was conducted (22). The two researchers conducted all the fieldwork and analysis to ensure the consistency and opportunities for cross-validation in relation to the collection and interpretation of data. Through reading the transcripts, researchers produced a summary of initial ideas, and discussed with each other to develop initial coding framework. Quotations, with minor changes to improve readability, have been extracted from the data where they gave a good example of a finding or captured what several participants said.

The qualitative software management system NVivo, version 11 (QSR International Inc., Burlington, MA, USA), was used to systematically organize the transcripts and to support coding and data analysis.

## 3. Results

### 3.1. Characteristics of participants

Characteristics of survey participants are presented in Table 1. Of the 2081 respondents with valid data, 62.37% lived in urban areas, and 81.45% of caregivers’ residence is registered locally. Over half (54.45%) caregivers had obtained junior college or bachelor level education or above, and 39.64% had an annual household income of less than CNY 50,000. Around 52% of caregivers’ children were male, and a majority (79.53%) were ≥ 24 months old.

**Table 1:** Characteristics of respondent in survey, *N* (%).

Characteristics	Total (2020), *N*(%)	Influenza vaccination during 2020–2021 flu season, *N*(%)	Total (2019), *N*(%)	Influenza vaccination during 2019–20 flu season, *N*(%)
Total	2081	43.63%	1796	46.10%
Region				
Anhui province	825 (39.64%)	398 (48.24%)	682 (37.97%)	369 (54.11%)
Shenzhen city	360 (17.29%)	134 (37.22%)	320 (17.82%)	133 (41.56%)
Shaanxi province	896 (43.06%)	376 (41.96%)	794 (44.21%)	326 (41.06%)
Living area				
Urban	1298 (62.37%)	592 (45.61%)	1116 (62.14%)	530 (47.49%)
Rural	783 (37.63%)	316 (40.36%)	680 (37.86%)	298 (43.82%)
Registered residence				
Local residents	1695 (81.45%)	750 (44.25%)	1488 (82.85%)	683 (45.90%)
Internal migrants	386 (18.55%)	158 (40.93%)	308 (17.15%)	145 (47.08%)
Caregiver’s age group (years)				
<=30	551 (26.48%)	247 (44.83%)	440 (24.50%)	208 (47.27%)
~35	880 (42.29%)	388 (44.09%)	757 (42.15%)	343 (45.31%)
~40	384 (18.45%)	164 (42.71%)	357 (19.88%)	173 (48.46%)
>40	266 (12.78%)	109 (40.98%)	242 (13.47%)	104 (42.98%)
Gender (Caregiver)				
Female	1672 (80.35%)	715 (42.76%)	1449 (80.68%)	651 (44.93%)
Male	409 (19.65%)	193 (47.19%)	347 (19.32%)	177 (51.01%)
Caregiver relationship with children				
Mother	1,621 (77.9%)	691 (42.63%)	1,410 (78.51%)	630 (44.68%)
Father	330 (15.86%)	153 (46.36%)	278 (15.48%)	141 (50.72%)
Grandparents and others	130 (6.25%)	64 (49.23%)	108 (6.01%)	57 (52.78%)
Education				
Middle school or below	501 (24.07%)	189 (37.72%)	444 (24.72%)	193 (43.47%)
High school	447 (21.48%)	185 (41.39%)	391 (21.77%)	178 (45.52%)
Junior college	537 (25.8%)	250 (46.55%)	455 (25.33%)	220 (48.35%)
Bachelor degree or above	596 (28.64%)	284 (47.65%)	506 (28.17%)	237 (46.84%)
Annual household income (1,000 Renminbi)				
<20	426 (20.47%)	172 (40.38%)	376 (20.94%)	149 (39.63%)
20–50	399 (19.17%)	172 (43.11%)	337 (18.76%)	153 (45.40%)
50–100	540 (25.95%)	253 (46.85%)	460 (25.61%)	235 (51.09%)
100–200	439 (21.1%)	196 (44.65%)	386 (21.49%)	189 (48.96%)
>200	277 (13.31%)	115 (41.52%)	237 (13.20%)	102 (43.04%)
Gender (Child)				
Female	996 (47.86%)	449 (45.08%)	871 (48.50%)	410 (47.07%)
Male	1085 (52.14%)	459 (42.30%)	925 (51.50%)	418 (45.19%)
Child’s age group (months)				
<24	426 (20.47%)	222 (52.11%)	644 (35.86%)	320 (49.69%)
> = 24	1655 (79.53%)	686 (41.45%)	1152 (64.14%)	508 (44.10%)

Among 38 caregivers who received interview: Most were women (23), aged 25–35 (24), educated to High school and below (15) (Table 2).

**Table 2:** Characteristics of caregivers participating in the interview.

Characteristics	Total, *n*
Total	38
Children’s influenza vaccination status in the 2020–2021 flu season	
Vaccination	17
Non vaccination	18
Non vaccination while with the willingness to receive vaccination	3
County/city	
Rural county, Anhui province	8
Urban city, Anhui province	10
Rural county, Shaanxi province	6
Urban city, Shaanxi province	6
Shenzhen city	8
Relationship with children	
Mother	32
Father	6
Age (years)	
25–30	5
30–35	19
35–40	9
>40	3
Education	
High school and below	15
Junior college	9
Undergraduate and above	13

### 3.2. Quantitative results on vaccination uptake

Of the 2081 respondents, a total of 1796 were in the age group for high-risk groups in the 2019–2020 flu season, and 46.10% reported that they vaccinated their children against influenza in the 2019–2020 flu season; meanwhile, 43.63% said that they vaccinated their children against influenza in the 2020–2021 flu season. In rural area, 43.82 and 40.36% of caregivers said that they vaccinated their children against influenza in the 2019–2020 and 2020–2021 flu season, while 47.49 and 45.61% indicated that they did in the 2019–2020 and 2020–2021 flu season in urban area. For caregivers with children younger than 24 months, 49.69 and 52.11% said that they vaccinated their children against influenza in the 2019–2020 and 2020–2021 flu season, while 44.10 and 41.45% of caregivers with children older than 24 months indicated that they did in the 2019–2020 and 2020–2021 flu season (Table 1).

### 3.3. Qualitative results on vaccination decision process and reasons

Of all caregivers interviewed, 17 vaccinated their children against influenza in the 2020–2021 flu season, 18 did not vaccinate their children and 3 were willing to vaccinate children but ultimately did not. Their vaccination decision process and reasons are as follows.

#### 3.3.1. Availability of influenza vaccination services

Most caregivers expressed satisfaction with the transportation to and from the vaccine clinics and the vaccination service provided by HCWs. Just over half of respondents reported no conflicts between their schedules and the time of vaccination services, a small number said they experienced time conflicts.

“Today is fine, (but) usually there will be many people waiting here, I might have to wait in line all morning, (and) because I own my own store, so I cannot open my store all morning” (Caregiver 28, female, Qindu District, Xianyang city).

Regarding the price of influenza vaccine ranging from 31 to 298 Chinese Yuan, the same number of respondents indicated that influenza vaccine was affordable.

“I think I can accept the price. Because we pay more attention to the health of children, not to the cost, we only value whether it has a protective effect on children, as long as it is beneficial to him, we will choose it. [..]” (Caregiver 22, female, Shushan District, Hefei city).

#### 3.3.2. Reduction in caregivers’ risk perception of influenza during COVID-19

Half of the caregivers indicated that the risk of influenza remained the same or decreased during the pandemic. Nearly half said that the adoption of nonpharmacologic interventions (NPIs), such as mandated face coverings in public, reduced the risk of getting the flu.

“It is certainly an effective protection. Double protection. In the past, people did not pay much attention to wearing masks and keeping distance with each other, but due to the COVID-19 pandemic, we pay more attention to these things, and the awareness of personal protection has been strengthened. We know it also has a positive effect in blocking this spread of influenza” (Caregiver 22, male, Shushan District, Hefei city).

#### 3.3.3. Low risk perceptions of influenza and high confidence in influenza vaccines

More than half of the caregivers indicated that their children were highly susceptible to influenza. However, only a few reported that getting the flu would lead to serious health consequences. Caregivers low level of perceived severity of influenza was associated with their understanding of the health consequences of influenza infection. Among all caregivers, half of them thought that influenza is an illness similar to a cold.

“The symptoms of the flu? Like catching are a cold, fever, a runny nose. That’s all I know. I really do not pay attention to the rest” (Caregiver 4, male, Nanshan District, Shenzhen city).

Most caregivers had a high level of confidence in the safety of influenza vaccines because they believe that government and the healthcare system were trustworthy, and they have strict oversight and testing of vaccine development and production. Some caregivers however remained uncertain about influenza vaccines’ effectiveness and importance. The main reason is that they believe the virus is constantly mutating, and the vaccine will not work against the mutated virus.

“Because flu virus mutates very fast, all of the vaccines are against influenza virus which have been discovered, if we have not found it yet, there is no effective vaccine. Now the speed of vaccine development cannot keep up with the speed of virus mutation. [..]” (Caregiver 5, female, Nanshan District, Shenzhen city).

We found that the caregivers’ perceived severity of influenza in children and confidence in influenza vaccines’ effectiveness and importance differed between participants who made different decisions about childhood influenza vaccination. Caregivers who vaccinated their children against influenza reported greater likelihood of perceiving high severity of influenza in children and more confidence influenza vaccines’ effectiveness and importance.

#### 3.3.4. Limited knowledge of influenza among caregivers and a lack of health information

The majority of caregivers in the three provinces indicated that they did not have a good knowledge of influenza. Their general perspectives are that influenza is an infectious disease, but the causes, symptoms and possible complications of influenza are not well understood.

“I do not know the cause of the flu, but I always feel that it is due to the weather or the infection? If you ask me which virus caused it? I do not know, it may be a cold” (Caregiver 17, male, Shushan District, Hefei city).

The reason why caregivers don not have a good knowledge of influenza may be that caregivers receive limited health information. Over half of participants said they did not have any access to health information. Less than half of caregivers said they had not been exposed to any flu-related information.

“The health information I am concerned about is mainly more in terms of parenting, sent by the official account in WeChat, or TikTok. Mainly about children’s education, and less information about vaccines. Because I have vaccinated my child (with) all the required vaccines, so there is nothing else to think about” (Caregiver 30, female, Qindu District, Xianyang city).

#### 3.3.5. Insufficient communication with professional information sources

Many caregivers reported lack of communication with HCWs about vaccination. Because professional information sources cannot meet the information needs of caregivers, some caregivers said that they will look for relevant health information through the Internet. At the same time, caregivers also said that HCWs will only briefly introduce the age-appropriate non-EPI vaccines to them when they take children to vaccination clinics. Caregivers understood the enormous daily workloads that the HCWs faced.

“Not a lot of communication. After all, there are a lot of people in the hospital. When you have questions for these healthcare workers, their answer is the same, just a few words, there is no in-depth talk. We do not have much access to doctors. I do not have much contact with them. [..]” (Caregiver 1, male, Nanshan District, Shenzhen city).

Over half of the caregivers said they had not received any flu vaccine-related health education from staffs in other medical institutions, including CHCs, and CDCs. The brochures and videos provided at vaccination clinics did not interest them and influence their decision on vaccination. Caregivers who indicated that they had received health education at vaccination sites said that the content of health education is mainly about vaccination procedures, vaccine types and how to deal with contraindications and emergency responses.

Another reason for insufficient communication between caregivers and HCWs is the vaccination schedules of EPI vaccines and non-EPI vaccines. When children are 1 year old, the number of EPI vaccines required on the vaccination schedule and the non-EPI vaccine greatly reduce, resulting in HCWs being unable to remind parents of the need for influenza vaccination face-to-face.

“I did not know there was such a thing as a flu shot. Every shot is indicated in the vaccine record book. My child needs to be vaccinated at the age of 6, but between the ages of 3 and 6, the record book is empty and says nothing about any vaccination. This is for you to choose. And then when the doctor tells you about it, they only tell you about EPI vaccine, so I did not pay attention to vaccinations for years. The flu vaccine is still a bit under-advertised” (Caregiver 24, female, Shushan District, Hefei city).

## 4. Discussion

Our study explored the reasoning behind decisions on childhood influenza vaccination during the COVID-19 pandemic. We found a similar coverage (around 45%) of influenza vaccination in the 2019–2020 and 2020–2021 flu seasons. Our study showed that many caregivers indicated that the adoption of NPIs during COVID-19 reduced the risk of influenza infection for children. We also found that there is limited knowledge about influenza and its potential severity among caregivers. It was influenced by inadequate communication between caregivers and professional information sources including HCWs and staffs from other medical institutions.

In our study, Influenza vaccination rates among children were higher than that before some years (13, 24–27). Influenza vaccination uptake was similar during the 2019–2020 and 2020–2021 flu seasons. Qualitative results show that many caregivers believed that the adoption of NPIs, such as use of face coverings and reinstating local lockdowns during COVID-19, reduced the incidence of influenza, and ultimately chose not to vaccinate their children against the flu. However, as restrictions on individual movement are loosened, the transmission on influenza is expected to increase (18). Hence, education campaign will be critical to address caregivers’ lack of risk perception on influenza during COVID-19.

Our study highlighted that caregivers think that children are highly susceptible to influenza, but that the consequences of infection are not serious, and this was also observed in other studies (13, 14). Perceived low severity of influenza may be linked to caregivers confusing the common cold with influenza and/or influenza-like illness (28). It resonates with previous study in Xining City, Qinghai province, which showed that 40% of parents of local kindergarten children perceived Influenza as a common cold (13). Meanwhile, caregivers surveyed in this study conveyed high level of confidence in the safety of influenza vaccine. Confidence in vaccine safety stems primarily from the public’s trust in government departments and HCWs. This trust is also the main reason that vaccine confidence among the public was restored relatively quickly after vaccine quality incidents in China (29–32). Despite evident confidence in the safety of influenza vaccine, we observed clear uncertainty about the effectiveness and importance of influenza vaccines in some caregivers. This is consistent with our quantitative study, which showed that while 84.72% of caregivers considered influenza vaccine safe, only 75.01% of caregivers considered influenza vaccine effective (20). The reason for the lack of confidence in the effectiveness of caregivers is the belief that the virus is constantly mutating so that the vaccine is not effective in preventing infection and not necessary. Despite strong clinical evidence demonstrating the efficacy of the influenza vaccine (23, 33–35), it can be speculated that the public is not clear about these data, which could in turn translates into a reluctance to vaccinate.

Although respondents had a clear perception about the severity of influenza and attitude toward influenza vaccine, as well as a fair bit understanding of the mutation of influenza viruses, we found that caregivers lack sufficient knowledge about influenza, including the causes, symptoms, and complications of the flu. Previous studies in China also showed a lack of knowledge about influenza among caregivers, with only 21.6% of parents saying they knew how influenza is transmitted (13). Our results suggested that their perceptions and attitude toward influenza and vaccine were not supported by scientific evidence. The study also showed that caregivers receive little influenza-related health information. Regarded as the most reliable information sources (36, 37), HCWs did not provide adequate information for caregivers’ influenza vaccination decisions. Many caregivers were only informed about the availability of influenza vaccines and did not have in-depth communication with HCWs, which resonates with previous qualitative research that found health professionals only provided information about non-scheduled vaccines to caregivers and emphasized parental autonomy in decision-making at the same time (38). Further research is needed to quantitatively describe how HCWs recommend non-EPI vaccines and figure out its influencing factors. Caregivers in our study also indicated that they had not received health education about vaccines from staffs in other medical institutions, including CHCs, and CDCs, or paid little attention to existing health education methods, such as brochures and videos. The results suggest that current methods for providing health information need to be reviewed and optimized. Over the last several decades, the practice of health communication has undergone significant changes, with increasing recognition that health decisions are shaped not only by knowledge or awareness but also by risk perception and self-efficacy (39). Therefore, in addition to simply describing knowledge, health communication programs need to make health information interpretable, persuasive, and actionable. To better motivate and change the public’s choices on vaccination, it is necessary to summarize the existing ways of health education and extract the effective parts to be implemented. In addition, to improve the accessibility of information for caregivers who cannot receive vaccination notifications without going to the vaccine clinics, medical institutions should make use of the Internet, including social media, to actively share vaccination-related information to caregivers in a timely manner and address caregivers’ misconceptions regarding immunization and influenza.

Our research shows that most caregivers were satisfied with the access of influenza vaccination services, while some caregivers were dissatisfied with the time of vaccination services and the price of influenza vaccines. Targeted interventions are necessary, including extending vaccination service hours and establishing more vaccine clinics close to residential areas. Furthermore, previous research showed that a subsidy that would reduce the price for caregivers could contribute to increasing the demand for non-EPI vaccination (40). Our results suggest that an expansion of financing sources is needed to alleviate economic barriers to the cost of influenza vaccines, and specific strategies should also be tailored to each region according to their disease burden and fiscal capacity.

### 4.1. Strengths and limitations

The main strength of our study was the nature of the mixed-methods study. The combination of questionnaire survey and semi-structured interviews could help to understand the changes in uptake rate of childhood influenza vaccination during the pandemic, as well as caregivers’ individual considerations regarding decisions of childhood influenza vaccination. In addition, the cooperation of local CHCs helped the researchers gain trust and acceptance among participants. There were several limitations to our study. Firstly, given the qualitative nature of the research, the participant sample size for our study limits its generalizability. A quantitative research design approach is needed to extrapolate the findings to the wider community. Secondly, sample bias is applicable to this study as participants showed a genuine interest in the management and prevention of respiratory tract infections in young children, which may not apply across all parental cohorts. Thirdly, our findings concerning other parties involved in health communication including the HCWs, staffs from CHCs and CDCs were based on indirect answers from caregivers. Further studies need to focus on HCWs responsible for providing or managing immunization services, who would likely have provided different and additional perspectives. Fourthly, we did not investigate the impact of insurance status of the respondents and their children on their decision on childhood influenza vaccination. Nonetheless, insurance status of caregivers have been shown to be not associated with caregivers’ vaccine hesitancy (29).

## 5. Conclusion

Our study reveals higher influenza vaccination uptake rates among children than previous studies. However, the adoption of NPIs during COVID-19 reduced the risk perception of influenza among caregivers. A number of caregivers had a poor knowledge of the influenza, and there were misconceptions that the influenza is not serious and that the flu vaccine is not effective. Many caregivers received little health information and had inadequate communication with professional information sources, including HCWs and staffs from other medical institutions. We also found that caregivers were generally satisfied with access to vaccine services, except for the price and where there were conflicts between their schedule and the time of vaccine services. More evidence-based interventions are needed to encourage communication between HCWs and caregivers, raise caregivers’ risk perception of influenza and eliminate misconceptions. In addition, extending vaccination service hours and increasing the number of vaccination clinics close to residential areas and expansion of financing sources for self-paid vaccination, could help improve access to vaccination services.

## Data availability statement

The raw data supporting the conclusions of this article will be made available by the authors, without undue reservation.

## Ethics statement

The studies involving human participants were reviewed and approved by the Fudan University School of Public Health, and the London School of Hygiene and Tropical Medicine Ethics committees (FDU IRB#2018-10-0703, LSHTMEthics Ref 16016). The participants provided their written informed consent to participate in this study.

## Author contributions

ZH, ST, TC, and HL designed the study. KH, QW, SH, YX, and JD collected the data. KH cleaned the data, performed the statistical computations, prepared the results, and drafted the manuscript. SH, TC, and HL critically edited the manuscript. All authors have reviewed, edited the manuscript, read, and agreed to the published version of the manuscript.

## Funding

This research was funded by the NIHR (16/137/109) using UK aid from the UK Government to support global health research. The funder had no role in any aspect of the study, other than funding the research collaboration that provided the financial resources to conduct the study.

## Conflict of interest

The Vaccine Confidence Project, which HL leads, receives collaborative grants with Astra Zeneca, GlaxoSmithKline, J&J, and Merck in addition to public sector grants.

The remaining authors declare that the research was conducted in the absence of any commercial or financial relationships that could be construed as a potential conflict of interest.

## Publisher’s note

All claims expressed in this article are solely those of the authors and do not necessarily represent those of their affiliated organizations, or those of the publisher, the editors and the reviewers. Any product that may be evaluated in this article, or claim that may be made by its manufacturer, is not guaranteed or endorsed by the publisher.

## Author disclaimer

The views expressed in this publication are those of the author (s) and not necessarily those of the NIHR or the UK government.
